# Decoding the Traumatic Memory among Women with PTSD: Implications for Neurocircuitry Models of PTSD and Real-Time fMRI Neurofeedback

**DOI:** 10.1371/journal.pone.0134717

**Published:** 2015-08-04

**Authors:** Josh M. Cisler, Keith Bush, G. Andrew James, Sonet Smitherman, Clinton D. Kilts

**Affiliations:** 1 Brain Imaging Research Center, Department of Psychiatry, University of Arkansas for Medical Sciences, Little Rock, Arkansas, United States of America; 2 Department of Computer Science, University of Arkansas at Little Rock, Little Rock, Arkansas, United States of America; Univ of Toledo, UNITED STATES

## Abstract

Posttraumatic Stress Disorder (PTSD) is characterized by intrusive recall of the traumatic memory. While numerous studies have investigated the neural processing mechanisms engaged during trauma memory recall in PTSD, these analyses have only focused on group-level contrasts that reveal little about the predictive validity of the identified brain regions. By contrast, a multivariate pattern analysis (MVPA) approach towards identifying the neural mechanisms engaged during trauma memory recall would entail testing whether a multivariate set of brain regions is reliably predictive of (i.e., discriminates) whether an individual is engaging in trauma or non-trauma memory recall. Here, we use a MVPA approach to test 1) whether trauma memory vs neutral memory recall can be predicted reliably using a multivariate set of brain regions among women with PTSD related to assaultive violence exposure (N=16), 2) the methodological parameters (e.g., spatial smoothing, number of memory recall repetitions, etc.) that optimize classification accuracy and reproducibility of the feature weight spatial maps, and 3) the correspondence between brain regions that discriminate trauma memory recall and the brain regions predicted by neurocircuitry models of PTSD. Cross-validation classification accuracy was significantly above chance for all methodological permutations tested; mean accuracy across participants was 76% for the methodological parameters selected as optimal for both efficiency and accuracy. Classification accuracy was significantly better for a voxel-wise approach relative to voxels within restricted regions-of-interest (ROIs); classification accuracy did not differ when using PTSD-related ROIs compared to randomly generated ROIs. ROI-based analyses suggested the reliable involvement of the left hippocampus in discriminating memory recall across participants and that the contribution of the left amygdala to the decision function was dependent upon PTSD symptom severity. These results have methodological implications for real-time fMRI neurofeedback of the trauma memory in PTSD and conceptual implications for neurocircuitry models of PTSD that attempt to explain core neural processing mechanisms mediating PTSD.

## Introduction

Posttraumatic Stress Disorder (PTSD) is characterized by re-experiencing of the traumatic event, avoidance of trauma-related stimuli, general changes in mood and cognition, and hyperarousal symptoms[1]. PTSD has a prevalence rate of ~8% [2] and is associated with markedly decreased quality of life and psychiatric and physical comorbidity [2,3]. Towards the larger goal of developing optimally effective interventions for PTSD, much research over the past two decades has focused on identifying the neurocircuitry mediating the core clinical symptoms of PTSD. Extant PTSD neurocircuitry models [4–8], derived from this large body of human neuroimaging and animal model research, emphasize altered functional activity of four neuroanatomical sites: the amygdala, hippocampus, ventromedial prefrontal cortex (vmPFC), and dorsal anterior cingulate cortex (dACC). Heightened activity of the amygdala and dACC are hypothesized to mediate the observed hyperarousal clinical symptoms (e.g., hypervigilance for threat, startle, etc.). By contrast, the vmPFC and hippocampus are hypothesized to mediate inhibition of the fear/traumatic memory and have been found to be hypoactive in PTSD, thereby ostensibly leading to re-experiencing and avoidance symptoms.

Meta-analyses that explicitly test the degree to which univariate functional activity within these regions is altered in PTSD provide partial support for these neurocircuitry models of PTSD. On the one hand, a recent fMRI meta-analysis [8] focusing on tasks probing generic emotional or cognitive constructs (i.e., not symptom provocation studies with ideographic trauma stimuli) demonstrated altered functional activation in PTSD in the amygdala, hippocampus, dACC, and vmPFC. On the other hand, this meta-analysis also found 1) that amygdala activity in PTSD patients was only hyperactive relative to non-trauma exposed controls and not to trauma-exposed controls, and 2) altered activity in numerous brain regions not specified by existing neurocircuitry models (e.g., lateral PFC, posterior cingulate cortex, etc). Similarly, a recent neuroimaging meta-analysis [9] of symptom provocation studies (i.e., trauma memory recall), which presumably model PTSD re-experiencing symptoms specifically, also suggested the additional involvement of the posterior cingulate cortex (pCC) and retrosplenial cortex and did not provide support for altered hippocampal or amygdala activity relative to control groups. These meta-analytic findings highlight the notion that while the neural regions implicated in the neurocircuitry of PTSD (amygdala, hippocampus, and medial PFC) are clearly important in PTSD, they are also clearly not sufficient to fully understand the neurocircuitry mediating all relevant clinical features of PTSD.

One approach towards clarifying the neurocircuitry mediating PTSD is multivariate pattern analysis (MVPA) [10–14]. In the traditional neuroimaging approach to understanding brain function, a psychological task is manipulated (e.g., viewing faces versus houses) and its effect on brain activity is observed. Analyses then test whether, across all stimulus presentations, a given region tends to be more active during one or the other stimulus class. In this approach, one is testing if knowledge of the stimulus provides predictive information about the brain response (e.g., given a face stimulus, one might expect fusiform gyrus activation). However, this link does not necessary extend in reverse; that is, it does not necessarily indicate that knowledge of a brain region’s activity is predictive about a stimulus. By contrast, MVPA explicitly tests this latter relationship and asks whether a given multivariate set of brain regions allows better-than-chance prediction of the generating stimulus. Additionally, whereas most prior PTSD-related neuroimaging research has focused on univariate analyses and identified functional activation of single regions (or at best functional connectivity between two regions), MVPA approaches seek to identify multivariate predictive relationships and can include the linear or non-linear simultaneous combination of all voxels in the brain. A focus on brain-wide functional interactions provides a notably better fit to the spatially distributed information processing that characterizes the human brain [15,16]. Accordingly, an MVPA approach might shed considerable light on the brain networks mediating PTSD. For example, a hypothesis derived from neurocircuitry models and recent meta-analysis is that symptom provocation tasks in PTSD (i.e., trauma memory recall) should concurrently engage a distributed network consisting of the amygdala, hippocampus, vmPFC, dACC, pCC, and retrosplenial cortex. This hypothesis can be tested stringently using an MVPA approach by testing whether the hypothesized multivariate set of brain regions reliably predicts trauma memory recall vs neutral memory recall. Further, prediction accuracy using the set of brain regions hypothesized in PTSD neurocircuitry models could be directly compared to alternative sets of brain regions (e.g., all grey matter voxels, all grey matter voxels excluding ROIs implicated in PTSD models, randomly generated ROIs, etc.) to test more stringently the hypothesis that the regions hypothesized by PTSD neurocircuitry models are indeed critical mechanisms mediating trauma memory recall in PTSD.

A related field that would be informed by clearer delineation of core neural mechanisms mediating observable PTSD clinical symptoms is real-time fMRI (rtfMRI) neurofeedback [12,17–20]. In rtfMRI neurofeedback, information about a participant’s brain state is presented back to that participant, in near real-time, allowing the participant to volitionally modulate that brain state. There are numerous examples in the literature of using rtfMRI neurofeedback. Perhaps most relevant to PTSD is recent research demonstrating that healthy and depressed individuals can be trained to increase amygdala activity in response to positively valenced stimuli through rtfMRI neurofeedback [17,21]. This body of data demonstrates that affected individuals can learn to volitionally control the neurocircuitry mediating their clinical symptoms. It would seem that efficacy of rtfMRI in reducing clinical symptoms is at least partially dependent upon targeting the correct neurocircuitry for the intended clinical symptoms. For example, if one were aiming to reduce distress upon intrusive recollections of the traumatic memory, one might expect that volitional control of the canonical regions implicated in PTSD (amygdala, hippocampus, vmPFC, dACC) would be helpful. In this manner, an MVPA approach towards a symptom provocation task (i.e., trauma memory recall) in PTSD would be informative regarding the multivariate set of brain regions that accurately predict trauma memory and therefore suggest the brain regions that might be targeted in rtfMRI neurofeedback for optimal clinical symptom reduction.

Here, we utilize a previously published data set [22] to conduct an MVPA of trauma memory recall among women with assault-related PTSD to 1) test whether a multivariate brain state can reliably predict trauma vs neutral memory recall, 2) define the methodological parameters that optimize classification accuracy, and 3) identify the correspondence between brain regions that differentiate trauma vs neutral memory recall and the brain regions predicted by neurocircuitry models of PTSD. It should be emphasized that the current lack of a trauma-exposed control group precludes inferences regarding whether brain regions encoding trauma memory recall differ among a PTSD sample. Nonetheless, the lack of a control group does not preclude inferences regarding whether the trauma memory can be reliably predicted based on brain state and whether the observed brain states correspond with neurocircuitry models. Further, individuals exposed to trauma who do not have PTSD exhibited resilience; thus, different brain states would be expected in this population and would not necessarily be informative regarding the brain states to target during rtfMRI for those who did develop PTSD.

## Method

### Participants and assessment

Participants consisted of 17 adult women with PTSD related to either physical or sexual assault. One woman moved excessively during the scan causing intractable signal artifact, and her data were subsequently removed from analyses. This resulted in a final sample of 16 participants. Inclusion criteria consisted of 1) a history of either physical or sexual assault, 2) a current diagnosis of PTSD, and 3) that participants were stable on any psychiatric medications for at least 4 weeks. Exclusion criteria consisted of psychotic disorders, a primary substance use disorder, or internal ferromagnetic objects. Participants were recruited from outpatient mental health clinics and from community wide advertisements. Table 1 lists demographic and clinical characteristics of this sample. All study procedures were approved by the University of Arkansas for Medical Sciences institutional review board and all participants provided written informed consent.

**Table 1:** Demographic and clinical characteristics of the 16 adult women in this sample.

Variable	Mean (or frequency)	*SD*
Age	33.8	10.8
Ethnicity	44% Caucasian	-
	50% African-American	
	6% Other	
Education	6% not graduate high school	-
	38% graduate high school or GED	
	38% some college	
	18% graduate 2 year college or more education	
Current Job	44% unemployed	-
Ever been hospitalized for psychiatric reason	38% yes	-
Age at first assault	9.53	6.66
% exposed to assault prior to age 18	75%	-
Age at last assault	26.19	10.49
% exposed to assault after age 18	75%	
PCL	62.3	13.5
BDI-II	24	11.7
Number of total direct assaults	7.6	2.4
Number of physical assaults from non-caregiver	3.0	1.2
Number of physical assaults from caregiver	1.6	1.2
Number of sexual assaults	2.9	1.4
Current Major Depressive Disorder	44%	-
Current PTSD	100%	-
Current Substance Use Disorder	25%	-
Medication	31% antidepressant (SSRI, SARI)	
	25% benzodiazepine	
	19% other (gabapentin, lamotrigine, zolpidem)	
	56% no medication	

Note. PCL = Posttraumatic Checklist—civilian version; BDI-II = Beck Depression Inventory-II.

The assault exposure histories of participants were characterized using the trauma assessment section of the National Women’s Survey and National Survey of Adolescents [23–25]. This is a structured interview used in prior epidemiological studies of assault and mental health functioning among adult women and adolescents. Specific assaultive events were assessed with behaviorally specific dichotomous questions and included: 1) sexual assault (i.e., anal penetration, vaginal penetration, oral sex on the perpetrator, oral sex from the perpetrator, digital penetration, fondling, forced fondling of the perpetrator), 2) physical assault (i.e., attacked with a weapon, attacked with a stick, club, or bottle, attacked without a weapon, threatened with a weapon, attacked with fists), and 3) severe abuse from a caregiver (i.e., beaten with fists or an object to the point where bruising or bleeding occurred).

PTSD and comorbid psychological disorders were assessed with the Structured Clinical Interview for DSM-IV Disorders (SCID) [26] administered by a trained clinical interviewer under the supervision of a licensed clinical psychologist. Participants additionally completed the Posttraumatic Stress Checklist-Civilian Version [27] and Beck Depression Inventory-II [28].

### Trauma Memory Recall Task

This task was delivered in the context of study probing the neural correlates of repeated exposure to the trauma memory [22]. Participants were first given a description and therapeutic rationale for repeated exposure to their traumatic memory (e.g., “coming in contact with the memory repeatedly allows you to process the memory and feel less distressed by it”). Participants then provided a written account of their primary assaultive event and also of a neutral control event. The narratives were collected with standardized methodology commonly used in the literature [29–34] that facilitates collection of relevant sensory detail (e.g., physical reactions, contextual stimuli, etc) during the event. The trauma and neutral scripts were matched in length (~3 min) and audiorecorded by a female research assistant.

Participants were asked to indicate their level of anxiety, vividness of the memory, and dissociation during the memory recall on Likert scales of 1–10, and practiced using this rating scale prior to fMRI. The imagery procedure during fMRI consisted of five sequential presentations of the memory. The narratives were presented both aurally through headphones and visually, such that the written narrative was presented to them on a projected screen that participants viewed through a mirror attached to the MRI head coil. Each three minute presentation was preceded by 30 seconds of a resting-state, and these 30 seconds were not used in the classification analyses described below. Participants provided the subjective ratings after each individual presentation of each narrative. The trauma and neutral narratives were presented sequentially (e.g., the neutral narrative was repeated five times, then the trauma narrative was repeated five times), with the order (neutral or trauma first) counterbalanced across participants. The two script types were separated by a 4 minute anatomical scan. Preliminary analyses demonstrated that anxiety during the first neutral script was not significantly higher among women who received the trauma scripts first compared to the women who received the neutral scripts first (*p* = .397).

### MRI acquisition

A Philips 3T Achieva X-series MRI system using an 8-channel head coil (Philips Healthcare, USA) was used to acquire imaging data. Anatomic images were acquired with a MPRAGE sequence (matrix = 256x256, 160 sagittal slices, TR/TE/FA = 2600ms/3.02ms/8°, final resolution = 1x1x1mm^3^ resolution). Echo planar imaging (EPI) sequences were used to collect the functional images using the following sequence parameters: TR/TE/FA = 2000ms/30ms/90°, FOV = 240x240mm, matrix = 80x80, 37 oblique slices (parallel to AC-PC plane to minimize OFC sinal artifact), slice thickness = 3 mm, final resolution = 3x3x3 mm^3^. Each memory presentation was collected as a separate EPI run (i.e., each participant had 10 runs for the 5 trauma and 5 neutral memory presentations).

### Image Preprocessing

Image preprocessing followed standard steps and was completed using AFNI [35] software. In the following order, images underwent despiking, slice timing correction, deobliquing, motion correction using rigid body alignment, alignment to participant’s normalized anatomical images, spatial smoothing using a 8 mm FWHM Gaussian filter (AFNIs 3dBlurToFWHM that estimates the amount of smoothing to add to each dataset to result in the desired level of final smoothing), and rescaling into percent signal change. Images were normalized using the MNI 452 template brain. Following recent recommendations [36], we corrected for motion related signal artifacts by using motion regressors derived from Volterra expansion, consisting of [R R^2^ R_t-1_ R^2^_t-1_], where R refers to each of the 6 motion parameters, and separate regressors for mean signal in the CSF and WM. This step was implemented directly after motion correction and normalization of the EPI images in the preprocessing stream. Grey matter (GM), CSF, and WM were segmented from the high resolution anatomical image using FSL

### Classification Analyses

#### General procedures

Across all methodological permutations tested, we used a common cross-validation procedure and classification algorithm. We used support vector machines (SVM) to train a classifier to discriminate trauma memory recall from neutral memory recall using the voxel-wise fMRI timeseries as the input features. The SVM algorithm used a non-linear radial basis function kernel and *C* parameter = 100. This algorithm attempts to find a decision boundary differentiating the classes of stimuli (trauma vs neutral memory recall) based on the nonlinear combination of features. SVM was implemented in Matlab using the LIBSVM library [37]. The features were the trial-by-trial % signal change (z-scored) values for each voxel within the specified brain mask (see below). We used 10-fold cross validation, such that the timeseries (e.g., 860 TRs) for a given participant was split into 10 equal segments (e.g., 86 TRs) of randomly chosen TRs. A classifier was trained using 9 of the segments and then tested on the left out segment, and this process was repeated until each segment was used as the left-out test sample. Classification accuracy was defined as the median accuracy across all 10 folds. The feature weight of each voxel was additionally stored for each cross-validation fold, and the final feature weight map was defined by each voxel’s median feature weight across the 10 folds.

#### Methodological permutations

We tested classification accuracy across the following permutations: 1) spatial smoothing vs no spatial smoothing, 2) number of memory recall repetitions (range 1–5), and 3) brain mask restricting which voxels are used in the SVM classifier (all GM voxels, only voxels within PTSD neurocircuitry ROIs, all GM voxels except voxels within PTSD neurocircuitry ROIs, only voxels within 5 randomly generated ROIs).

#### Spatial smoothing vs no spatial smoothing

Among datasets that were not spatially smoothed, the last step of the preprocessing pipeline was detrending, whereas among spatially smoothed data sets an additional spatial smoothing step (using an 8mm FWHM kernel; 3dBlurToFWHM) was included.

#### Number of memory recall repetitions

All participants underwent 5 successive memory recall repetitions for each script type. We tested classification accuracy and reproducibility of the SVM feature weight spatial maps between models trained using the first run of each script type, the first two consecutive runs, the first three consecutive runs, the first four consecutive runs, and all five runs.

#### Brain mask restricting which voxels are used in the SVM classifier

We compared classification accuracy between models trained using all GM voxels, only voxels within ROIs specified in PTSD neurocircuitry models, all GM voxels except those voxels within ROIs specified in PTSD neurocircuitry models, and only voxels within randomly generated ROIs. The GM mask was defined by segmenting each subject’s high resolution T1 image (using FSL) into GM, white matter, and CSF, and then selecting those GM voxels that are shared by at least half of the participants. This resulted in a common GM mask used across participants consisting of 36280 voxels.

As indicated in the introduction, all current neurocircuitry models of PTSD implicate the amygdala, hippocampus, and medial PFC as key neural mechanisms mediating PTSD, and a recent meta-analysis additionally implicated the retrosplenial cortex, PCC, and precuneus in PTSD re-experiencing symptoms specifically. The specific anatomical coordinates of the PTSD ROIs used here were derived empirically from two recent meta-analysis of functional activation studies in PTSD [8,9]: left amygdala (center-of-mass (COM) X,Y,Z coordinates = -20, -8, -16); left (COM X,Y,Z coordinates = -32, -20, -8) and right (COM X,Y,Z coordinates = 28,-12,-24) hippocampus; perigenual ACC (COM X,Y,Z coordinates = -10,24,22); dorsal ACC (COM X,Y,Z coordinates = 3,26,21); retrosplenial cortex (COM X,Y,Z coordinates = 0,-53,8), pCC (COM X,Y,Z coordinates = 2,-50,22), and precuneus (COM X,Y,Z coordinates = -1,-56,35). We placed 6mm spherical ROIs centered on each of these coordinates. Given that a 6mm ROI centered on the left amygdala coordinates, derived from the Patel et al. meta-analysis [8], encompasses the junction of the posterior amygdala and anterior hippocampus, we refer to this seed region as amygdalohippocampal throughout the manuscript. Voxels within these ROIs (and within the common GM mask described above) were then used to train the SVM classifier (total voxels = 243).

To foster comparisons regarding the explanatory power of the PTSD ROIs, we also compared classification accuracy to two control masks: 1) a mask using all GM voxels except for those implicated in PTSD neurocircuitry models (total voxels = 36037), and 2) voxels within 8 randomly generated ROIs (33 spherical voxels) within the GM mask (total voxels = 229). Regarding the randomly generated ROIs with the GM mask, we generated 10 sets of 8 randomly generated ROIs (all sets of ROIs available for inspection upon request from the first author), and tested classification accuracy for each participant using each of the 10 sets of random ROIs, and then defined classification accuracy for the random ROIs as the median cross-validation accuracy across all 10 random sets of ROIs.

#### SVM feature weight spatial maps reproducibility

Spatial maps representing each voxel’s contribution to the nonlinear SVM decision function were created for each participant across each relevant methodological permutation by reshaping the SVM feature weights back into 3-D brain shape. Reproducibility of the maps across methodological permutation was assessed by examining the spatial correlation (after r-to-z transformation to improve normality) between feature weights reshaped as 1-D vectors for a given participant. For example, for a given participant, we characterized the correlation of the SVM feature weights for the model trained using only the first run to the model trained using the first two runs.

#### Similarity of SVM feature weight maps across participants

We also sought to assess whether there were commonalities in the feature weights contributing to the nonlinear SVM decision function across participants. This was assessed in two ways. First, we assessed univariate similarity for each voxel within the GM mask across participants using traditional mass univariate one-sample t-tests. We similarly used mass univariate robust regression analyses to test whether SVM feature weights scaled linearly with PTSD symptom severity (from the PCL) when controlling for age, comorbid depression, and comorbid substance use disorders. We corrected for multiple comparisons using cluster-level thresholding, with a corrected p < .05 achieved through a minimum of 47 contiguous voxels surviving an uncorrected p < .01 (based on 3dClustSim). Second, to maximize statistical power, we conducted parallel one-sample t-tests and robust regression analyses with PTSD symptom severity when constraining analyses within the 8 PTSD-related ROIs described above. The mean SVM feature weight among the voxels within these ROIs was calculated for each participant and then used in subsequent one-sample t-tests or robust regression analyses.

#### Control classification analysis

Finally, we also conducted a control classification analysis to demonstrate that classification results and feature weight maps for differentiating trauma vs neutral memory recall cannot be explained by differences in motion-related artifact. In this analysis, we used framewise displacement (FD) to define motion across the 6 directions of motion displacement. FD refers to the sum of the absolute value of temporal differences across the 6 motion parameters; for example, an FD of .5 indicates a TR where the participant moved, in total across the 6 parameters, 5 mm. We used a median split to divide the FD into high motion TRs and low motion TRs, which were then the labels used in classification. The SVM classification analyses used the first three runs to parallel the analyses that seemed most efficient and accurate (see results below) for differentiating trauma vs neutral memory recall; thus, the FD were concatenated across the three runs prior to the median split. The SVM classification analyses used all GM voxels and were identical, except for the labels, to the corresponding three run model differentiating trauma from neutral memory recall.

## Results

### Effect of spatial smoothing and memory recall repetitions

**Fig 1:**
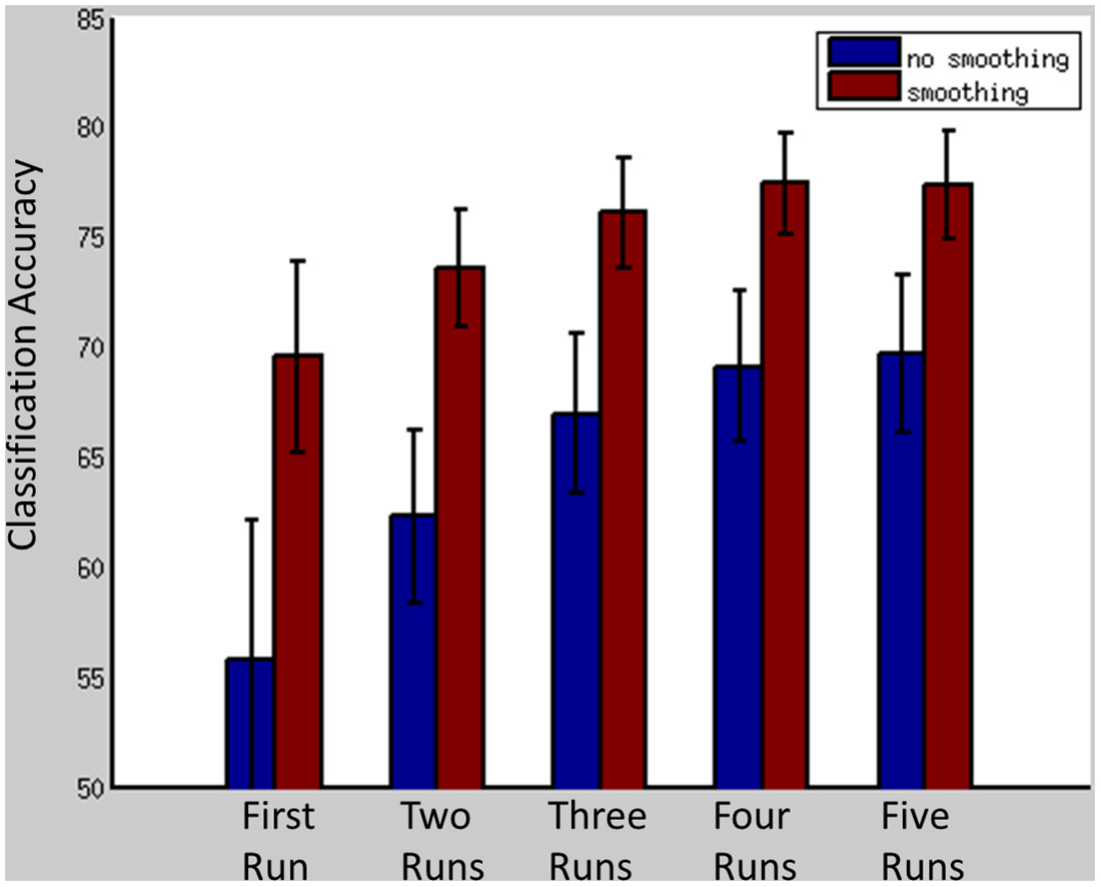
Mean classification accuracy across participants (and standard error) as a function of number of runs used to train the model and spatial smoothing.

Fig 1 depicts classification accuracy as a function of smoothing and number of memory recall repetitions. As can be seen, with the exception of non-smoothed data sets using only the first memory recall run, all other permutations provided classification accuracy significantly above chance levels. Classification accuracy was significantly better for smoothed vs unsmoothed data sets regardless of memory recall repetitions. When using smoothed data sets, two recall repetitions was not significantly better than just using the first run (p = .18), but using three runs, four runs, and five runs were all significantly better than using two runs (all *p*s < .05). Using four runs and five runs was not significantly better than three runs. As depicted in S1 Fig, classification accuracy for all participants was better than 60% when using at least two runs for model training.

### Reproducibility of SVM feature weight spatial maps

**Fig 2:**
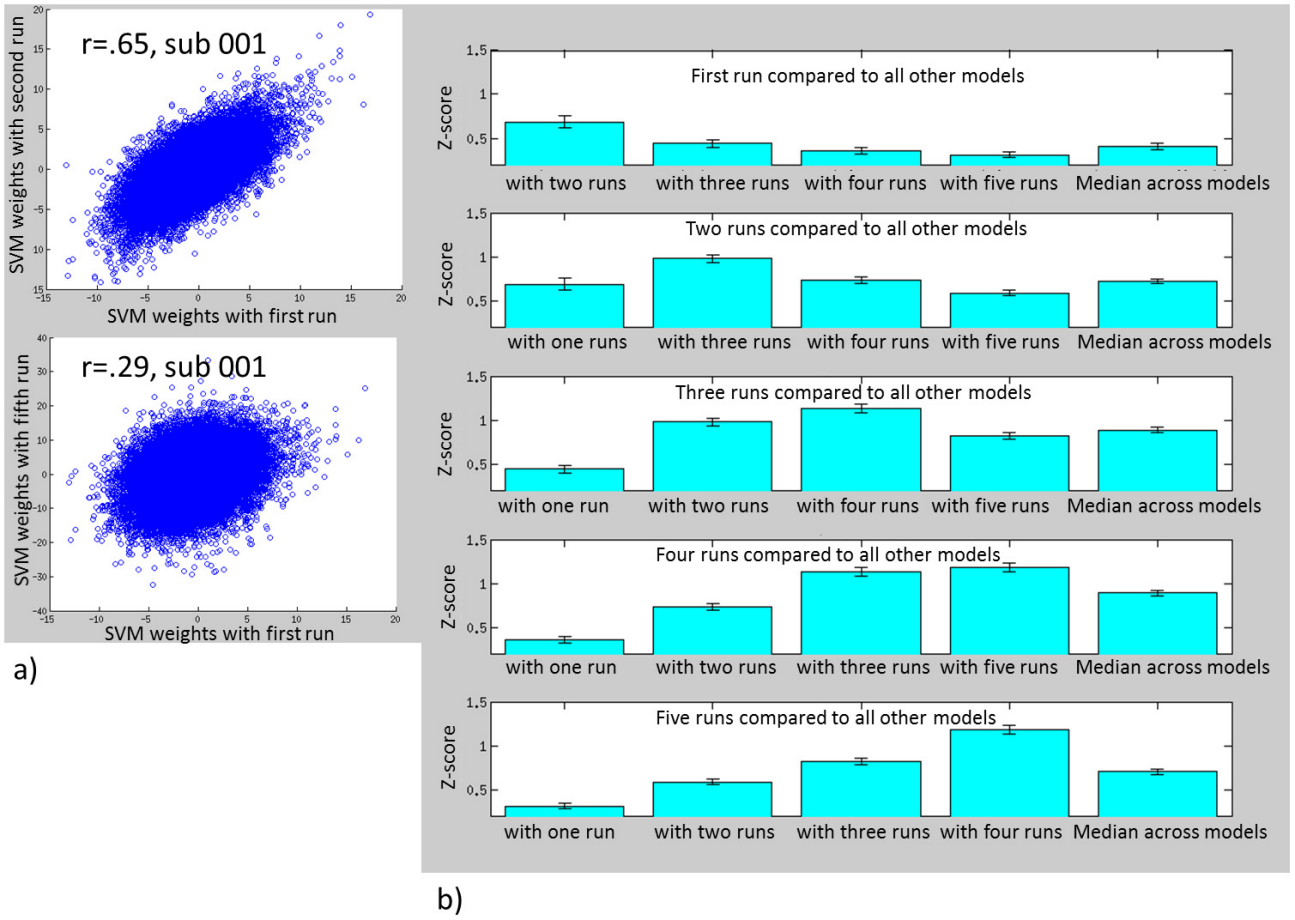
a) scatter plots for a single participant showing the spatial correlation between SVM feature weights for each voxel when trained using a single run with SVM feature weights when trained using two runs (top) and correlation between SVM feature weights when trained using a single run and SVM feature weights when trained using 5 runs. b) spatial correlation across participants for the voxelwise SVM feature weights between the different models.

We then assessed how similar (using r-to-z transformed correlation coefficients) the SVM feature weights were for a given participant across the models using different numbers of memory recall repetitions. Fig 2A depicts this approach for a single participant. This participant’s SVM feature weights across GM voxels for the model trained using the first run was highly similar to the SVM feature weights for the model trained using the first two runs (*r* = .65); by contrast, similarity between SVM feature weights for the model trained with the first run and the model trained with all five runs for this same participant was less robust (*r* = .29). When this approach is repeated across all participants (Fig 2B), SVM feature weights trained with 3–4 memory repetitions demonstrated greatest similarity across models. This was statistically tested by examining the median similarity for each model type (e.g., for the three run model, the median similarity of the three run model with all other models), which demonstrated that the median similarity across models for the model trained with the just the first run was significantly less than the similarity across models for the two run model (p < .001). The two run model had significantly less similarity across models than the three run model (p< .001), the three run model overall similarity did not differ from the four run model similarity (p = .97), and the four run model overall similarity was significantly greater than the five run model overall similarity (p < .001).

Combined these results suggest that the three and four run models had the greatest classification accuracy and yielded SVM feature weights with the greatest reproducibility. Given that repeated presentation of the trauma memory yields non-stationary brain dynamics, it seems that efficiency, reproducibility, and accuracy is best for the three run model. Thus, subsequent analyses are constrained to just models trained with three memory recall repetitions.

### Sensitivity and Specificity

Beyond classification accuracy (hit rate), we also assessed sensitivity (proportion of trauma narrative TRs correctly identified as trauma narrative TRs) and specificity (proportion of neutral narrative TRs correctly identified as neutral narrative TRs) of the three run model. Mean specificity across the 16 participants was 76%; mean sensitivity was 74%. There was no significant difference between sensitivity and specificity (*t* = .82, *p* = .42), suggesting that the classifier was not biased towards decisions of trauma or neutral memory recall.

### Effect of brain mask

**Fig 3:**
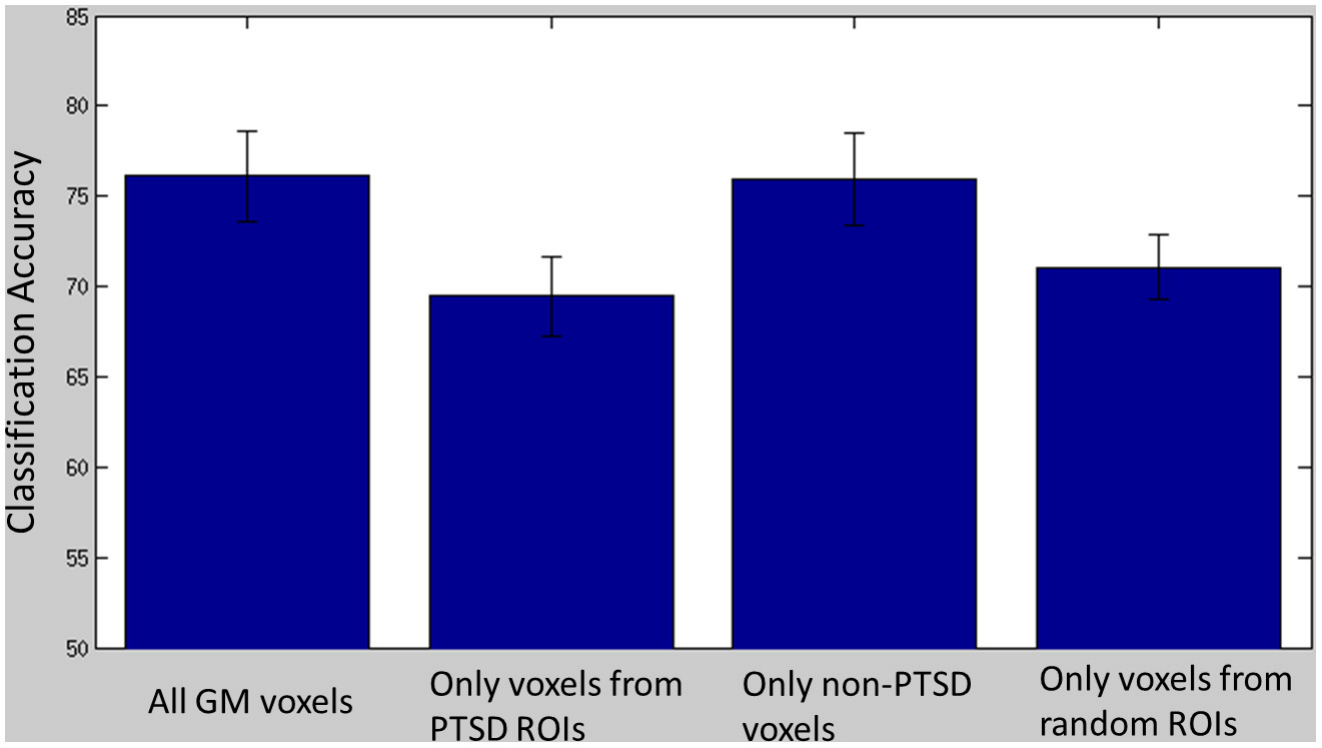
Mean classification accuracy across participants, trained using three runs, as a function of the brain mask used to select voxels: all GM voxels, only voxels within PTSD-related ROIs, all GM voxels except the PTSD-related voxels, and voxels within randomly generated ROIs.

Fig 3 depicts classification accuracy as a function of the voxels used to train the model when using three memory recall repetitions. Classification accuracy was significantly greater when using all GM voxels compared to the model using only voxels within the ROIs implicated in PTSD neurocircuitry models (p < .001) and the randomly generated ROIs (p < .001). Similarly, classification accuracy was significantly greater when using all GM voxels except those within ROIs implicated in PTSD neurocircuitry models compared to using only voxels within ROIs implicated in PTSD models (p < .001) and the randomly generated ROIs (p < .001). Finally, classification accuracy when using voxels only within ROIs implicated in PTSD models did not significantly differ from using only voxels within 8 randomly generated ROIs (p = .08).

### Similarity of SVM feature weight maps across participants

**Fig 4:**
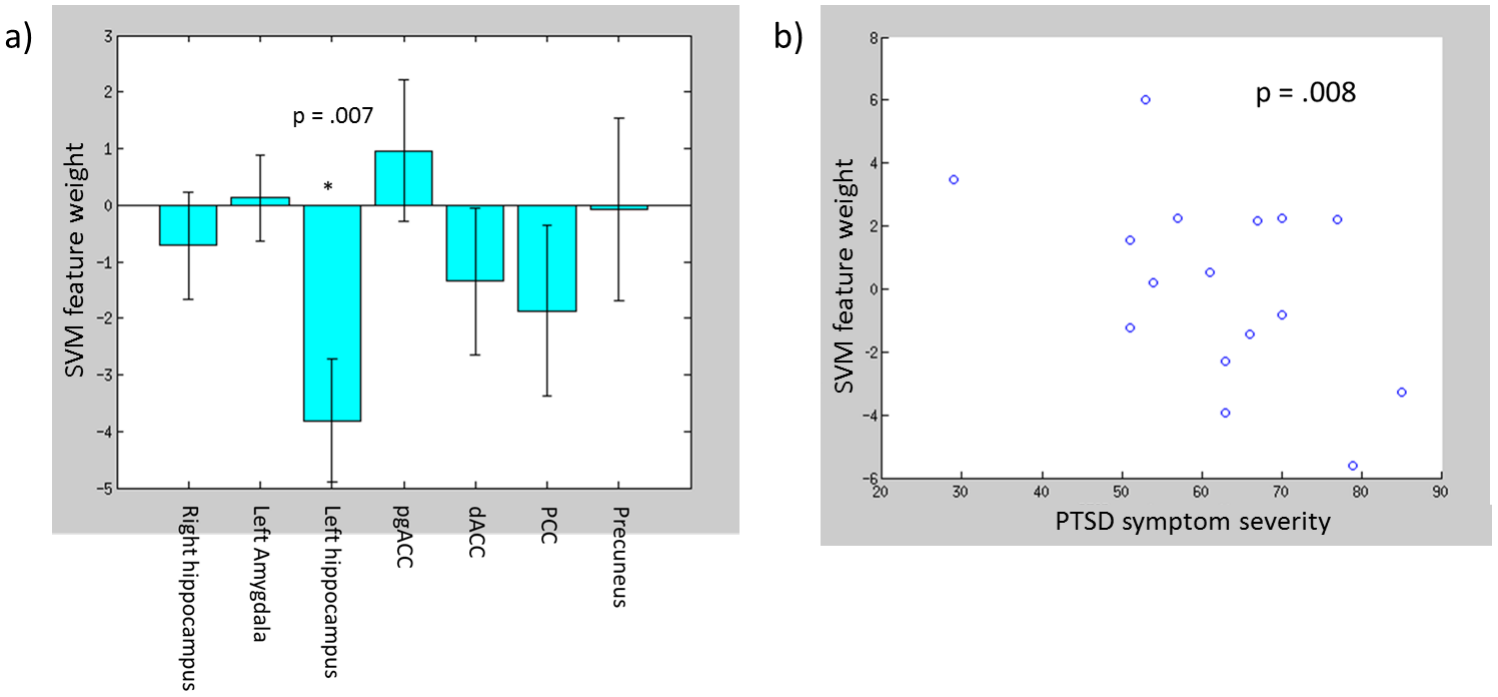
a) Mean SVM feature weights across participants with PTSD-related ROIs. b) scatter plot depicting negative linear relationship between PTSD symptom severity and mean SVM feature weight within the left amygdalohippocampal (p value comes from robust regression analysis also controlling for age, comorbid depression, and comorbid substance use disorders).

Voxelwise univariate analyses within the GM mask failed to identify significant clusters of voxels where the SVM feature weights either differed significantly from zero or significantly scaled with PTSD symptom severity. However, the ROI analyses (Fig 4A) demonstrated that the contribution of the left hippocampus to the SVM decision function was generally consistent across participant (i.e., its mean SVM feature weight across participants differed significantly from zero) (p = .007). Similarly, the ROI analyses demonstrated that the contribution of the left amygdalohippocampal ROI to the SVM decision function scaled linearly with the severity of PTSD symptoms (p = .008, when controlling for age, comorbid depression, and comorbid substance use disorders) (Fig 4B).

### Control Classification Analyses

**Fig 5:**
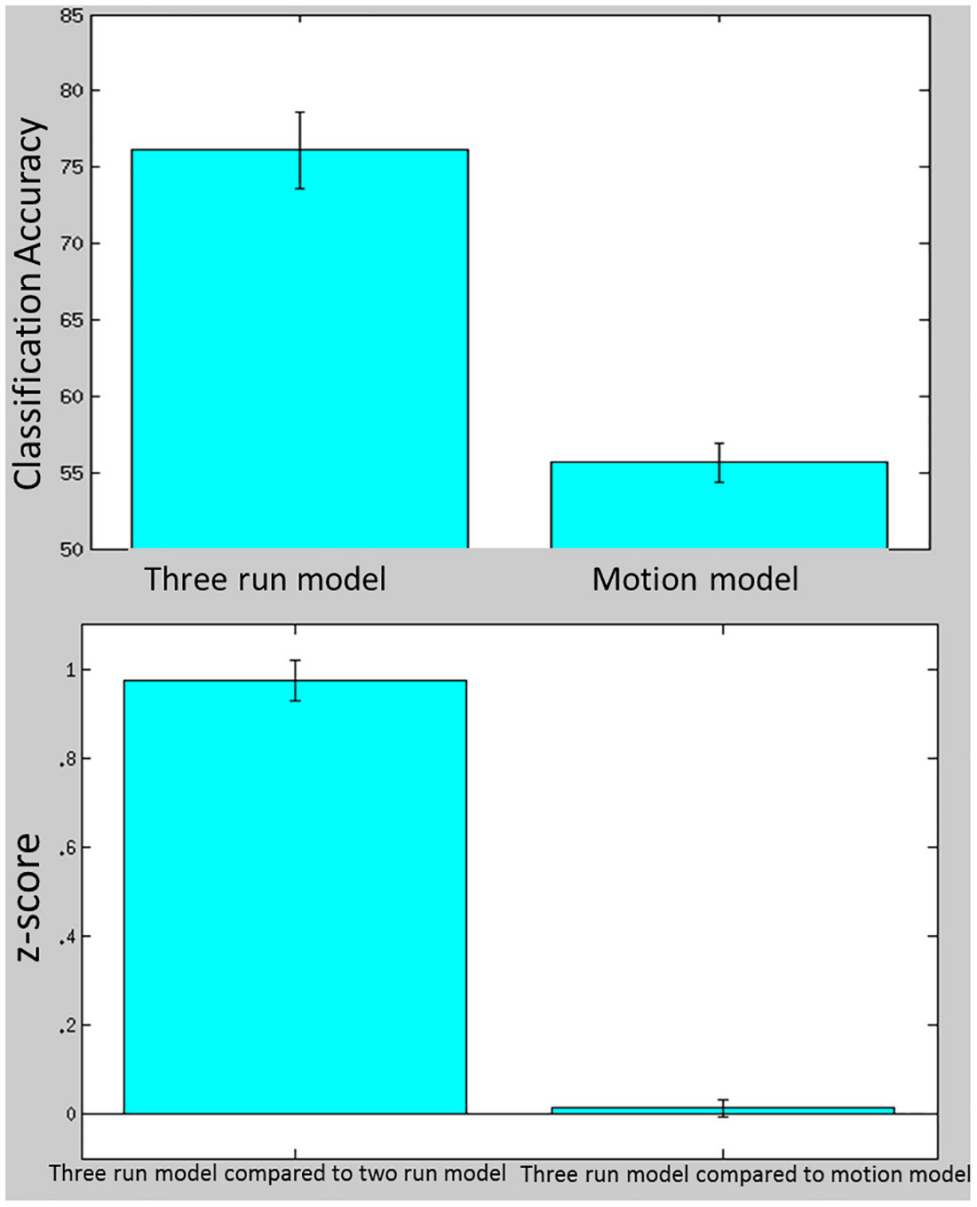
top) Mean classification accuracy across participants for the three run model and the model trained to differentiate high- and low-motion TRs. Bottom) Spatial correlation between the three and two run models trained to differentiate trauma from neutral memory recall, and spatial correlation between three run model trained to differentiate trauma recall and three run model trained to differentiate motion.

As indicated in Fig 5, mean classification accuracy for the three run model differentiating trauma vs neutral memory recall (M = 76.1%, SD = 10%) was significantly greater than the three run model differentiating high vs low motion TRs (M = 55.7%;SD = 5%); *t* = 7.2, *p* < .001. Similarly, there was significantly greater spatial correlation between the three run model feature maps and two run model feature maps differentiating trauma vs neutral memory recall compared to the spatial correlation of the feature maps between the three run model differentiating memory recall and three run model differentiating motion (*t* = 16.35, *p* < .001).

## Discussion

The purpose of the present study was to test 1) whether a multivariate set of brain regions could reliably discriminate between trauma and neutral memory recall among women with PTSD, 2) the methodological parameters that affect this classification accuracy, and 3) the correspondence between the brain regions that differentiate trauma recall and the brain regions predicted by PTSD neurocircuitry models. In regards to the first goal, we found that trauma memory recall could be differentiated reliably from neutral memory recall across women with PTSD based only on a snapshot (a single TR) of brain function. Mean classification accuracy across participants ranged from ~69%-77% for spatially smoothed data sets and were all significantly greater than chance. These basic results demonstrating significantly reliable classification accuracy have general implications. These data demonstrate that it is possible, and statistically reliable, to map a given individual with PTSD’s brain network encoding trauma memory recall. In a traditional univariate analyses, one would assess the statistical relationship between the task (e.g., trauma vs neutral memory recall) and the activity in a voxel, and this is repeated across all voxels. While this approach identifies the effect of the task on individual voxel activity, it does not provide information regarding 1) co-activity across spatially distributed voxels (i.e., a network level analysis), nor 2) the predictive validity of a voxel’s or set of voxels activity in differentiating the task. By contrast, these present results demonstrate both the spatially distributed patterns of voxel co-activity related to trauma memory recall and also the predictive validity of this network in differentiating trauma from neutral memory recall. Accordingly, the current study provides proof-of-concept evidence for applications of real-time fMRI neurofeedback to identify, and then target for volitional control, a brain-wide network encoding the trauma memory identified specifically for each individual.

In regards to the methodological parameters that optimize classification accuracy, we observed that spatial smoothing had a marked advantage over non-spatial smoothing. This is counterintuitive in the sense that most MVPA analyses use nonsmoothed data sets and a putative strength of MVPA is utilizing spatial information that is lost during spatial smoothing [38–41]. Nonetheless, spatial smoothing is also widely known to increase statistical power to detect univariate activations [38] and two separate studies have shown that spatial smoothing does not hurt, and might actually increase, the accuracy of MVPA classification analyses [42,43]. While the current study spatially smoothed with a uniform 8mm kernel across the brain, it is important to note this effectively lowers spatial resolution and affects interpretation of smaller subcortical structures, such as the amygdala or hippocampus. Future research focused on classification of trauma recall brain states or real-time neurofeedback might benefit from additional research testing the impact of the extent of spatial smoothing on classification accuracy and/or neurofeedback efficacy.

We also observed that using data from multiple runs was superior to using a single run, and that there appeared to be diminishing returns of increasing the number of runs after three runs. Beyond the observation that more data is clearly helpful in training the classifier to identify and separate the signal of interest, it is informative that increasing the number of runs beyond three does not improve classification performance. As noted above, the previous study from which these data come [22] demonstrated that neural networks during repeated exposure to the traumatic memory are not stationary; thus, for the purpose of mapping a given individual’s brain network encoding the trauma memory, one would need enough data to ensure accurate model building but one would not want to acquire so much data that one begins to change the network being targeted. In further support of this, we observed a decreased spatial correlation between the SVM feature weights of the one run model and models using more than two runs. Similarly, we also observed decreased spatial correlation between SVM feature weights of the five run model and models using less than four runs. By contrast, models trained using three runs and four runs appeared to retain greatest similarity across all models. Overall, these data suggest that spatial smoothing in this context is beneficial for classification accuracy, and that there is a trade-off between acquiring enough data for accurate model building and acquiring too much data that alters the structure of the network being identified.

We also investigated the correspondence between brain regions differentiating trauma from neutral memory recall and the brain regions predicted by PTSD neurocircuitry models. We found that a whole-brain approach (all GM voxels) was superior to an ROI approach. Further, while we found better than chance classification accuracy when using only voxels within PTSD neurocircuitiry-related regions, we also found that classification using PTSD neurocircuitry-related regions was no better than randomly generated ROIs, and that removing voxels with PTSD neurocircuitry-related ROIs did not weaken performance when using all other GM voxels. These results provide strong evidence that the brain networks encoding recall of the trauma memory cannot be reduced to regions canonically implicated in PTSD; rather, these data emphasize that distributed and brain-wide information processing mediates trauma memory recall. The GM voxel-wise group-level analyses failed to identify significant clusters of voxels where the SVM feature weights were similarly strong across individuals; however, the ROI analyses focused on PTSD neurocircuitiry-related ROIs demonstrated that the feature weights for the left hippocampus significantly differed from zero, and that SVM feature weights for the left amygdalohippocampal ROI were significantly negatively correlated with PTSD symptom severity. It is widely known that interpretation of SVM feature weights when using a non-linear kernel is difficult [12,44]. Nonetheless, it is interesting that hippocampal activity was reduced during the trauma memory (negative SVM weight) and that PTSD symptom severity was negatively related to SVM weight of the amygdalohippocampal (greater PTSD was associated with more negative SVM weight) in light of prior research demonstrating reduced limbic activity during acute stress exposure [45]. However, the analyses manipulating which voxels were used in the classification analyses demonstrates that the PTSD-related ROIs are useful (classification accuracy differed from chance when using them) but not necessary (removing them did not affect classification accuracy). Overall, the current results highlight the need to further define the brain-wide network organization, and their disruptions, that mediate trauma memory recall in PTSD.

The current results have implications for the use of rtfMRI neurofeedback in the treatment of PTSD. Based on prior meta-analyses of either generic cognitive-emotional tasks or symptom provocation specifically [8,9], one might predict that training individuals to decrease amygdala (or increase pgACC) activity would be helpful in modulating putative downstream re-experiencing symptoms. The current results suggest that targeting the individual regions implicated in PTSD neurocircuitry models would have only limited efficacy, as these brain regions have only limited predictive validity in discriminating trauma memory recall. Instead, the current results suggest that it might be more efficacious to build an idiosyncratic brain-wide map and train an individual to modulate this entire network. Indeed, it has previously been argued [12] that an idiosyncratic brain-wide approach has the advantages of 1) better matching the distributed information processing patterns in the brain, 2) being less dependent on inter-subject variability in cognitive strategy or anatomy, and 3) being more robust to fMRI signal challenges. In support of the feasibility of modulating an entire brain map, as opposed to an individual ROI, previous research has successfully trained individuals to modulate brain-wide maps identified during motor tapping, mood induction, language, and covert counting tasks [13,46]. rtfMRI-based neurofeedback in PTSD might then entail a series of training runs, where the patient undergoes at least two trauma and neutral memory recall repetitions that is used to build a whole-brain model of that individual’s brain network encoding of the trauma memory. The testing run would then entail providing some type of visual feedback to the patient about the activation of this brain network and allowing the individual to learn to modulate the level of activation. Once mastery is built in modulating the brain network, one might then complete additional trauma recall repetitions with concurrent neurofeedack to train the individual to modulate the brain network during recall of the trauma memory. A design that includes sham neurofeedback would be helpful in testing the efficacy of rtfMRI-based neurofeedback in reducing clinical symptoms. However, there is an important caveat to a brain-wide approach for rtfMRI neurofeedback: it is difficult to determine if any region or sets of regions are adaptive (e.g., regulatory) or dysfunctional. As such, it is not necessarily clear what functional impact training individuals to modulate the entire network would have. By contrast, an ROI-based approach might foster clearer interpretation of the specific function that is being trained with the feedback. This is an issue that can be addressed empirically with a clinical trial that randomly assigns individuals to ROI-based vs brain-wide rtfMRI neurofeedback and differences in efficacy and impact on function outcomes between the approaches can be directly compared.

The present results demonstrate the feasibility of using MVPA to identify whole-brain networks that differentiate trauma from neutral memory recall among women with PTSD. We also demonstrate the methodological parameters that seem to optimize classification accuracy, with spatial smoothing and repeated memory presentations (with diminishing returns after a certain point) emerging as important factors affecting accuracy. We also found that whole-brain networks, rather than PTSD-specific ROIs, led to optimal classification accuracy, which emphasize the distributed nature of trauma memory recall and highlight that re-experiencing symptoms cannot be reduced to a few ROIs. These results also have practical implications for application of rtfMRI neurofeedback in PTSD. Nonetheless, the current study is not without limitation. First, this was an offline MVPA analysis, and thus any speculation regarding neurofeedback efficacy, with respect to either modulating a whole-brain vs ROI target or modulating clinical symptoms, is purely speculative. Second, the sample was limited to a small sample of women with assault-PTSD. The focus on women with assault-related PTSD makes the degree to which these results generalize to other samples of PTSD (e.g., men, other types of trauma, etc) is unknown, and the small sample size accordingly tempers inferences and calls for additional research to replicate and extend the current findings. Similarly, the lack of a trauma-exposed group without PTSD precludes any inferences regarding the degree to which the identified brain regions are unique to PTSD vs common to trauma-exposure per se cannot be made. While this is a limitation in making inferences regarding whether the neurocircuitry in PTSD differs from trauma-exposed without PTSD, note that this is not a limitation regarding the application of MVPA to the problem of differentiating trauma memory recall in PTSD. In light of these limitations, future work along these lines is needed to further probe the brain networks that mediating trauma memory recall in PTSD, the unique differences in these networks among PTSD samples (vs trauma-exposed controls), the methodological factors that optimize identification of the brain networks, and the efficacy of rtfMRI-based neurofeedback in modulating these networks and downstream clinical symptoms.

## Supporting Information

S1 Fig Histogram showing the distribution of classification accuracy across the 16 participants as a function of number of runs used to train the model. (TIF)
