# Supplementary figures and images for: Decoding the Traumatic Memory among Women with PTSD: Implications for Neurocircuitry Models of PTSD and Real-Time fMRI Neurofeedback
Source: PLoS One. 2015 Aug 4;10(8):e0134717. doi: 10.1371/journal.pone.0134717 (PMC4524593; doi:10.1371/journal.pone.0134717)

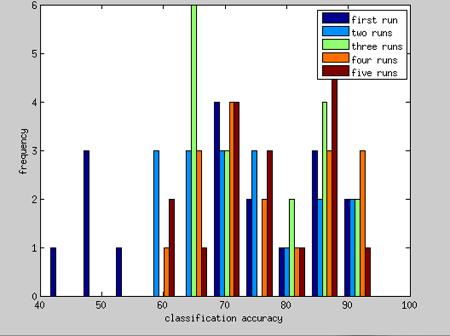

Supplement: S1 Fig — (TIF) [file pone.0134717.s001.tif]
